# Elderly versus non-elderly patients undergoing surgery for left-sided native valve infective endocarditis: A 10-year institutional experience

**DOI:** 10.1038/s41598-020-59657-1

**Published:** 2020-02-14

**Authors:** Chun-Yu Lin, Cheng-Hui Lu, Hsiu-An Lee, Lai-Chu See, Meng-Yu Wu, Yi Han, Chi-Nan Tseng, I-Li Su, Han-Yan Li, Feng-Chun Tsai

**Affiliations:** 1 Department of Medicine, College of Medicine, Chang-Gung University, Taoyuan, Taiwan, ROC; 2 Department of Cardiothoracic and Vascular Surgery, Chang-Gung Memorial Hospital, Linkou Medical Center, Taoyuan, Taiwan, ROC; 3 Department of Cardiology, Chang-Gung Memorial Hospital, Linkou Medical Center, Taoyuan, Taiwan, ROC; 4 Department of Public Health, College of Medicine, Chang-Gung University, Taoyuan, Taiwan, ROC; 5 Biostatistics Core Laboratory, Molecular Medicine Research Center, Chang-Gung University, Taoyuan, Taiwan, ROC; 6 Division of Rheumatology, Allergy and Immunology, Chang-Gung Memorial Hospital, Linkou Medical Center, Taoyuan, Taiwan, ROC

**Keywords:** Interventional cardiology, Valvular disease, Bacterial infection

## Abstract

This retrospective study aimed to clarify the short- and mid-term outcomes of elderly patients who underwent surgery to treat left-sided native valve infective endocarditis (LSNIE). Between July 2005 and September 2015, 179 patients underwent surgical treatment for active LSNIE at a single institution. Patients were classified into two groups: ≥65 years (elderly group) and <65 years (non-elderly group). Clinical features, surgical information, postoperative complications, and three-year survival rates were compared. The average ages were 74.2 ± 6.4 and 45.2 ± 12.6 years in the elderly and non-elderly groups, respectively. The elderly group had a higher predicted mortality rate and a lower incidence of preoperative septic emboli-related complications. Echocardiographic assessments of infected valves were generally homogenous between the groups. The elderly patients had a higher in-hospital mortality rate than the non-elderly patients (26.3% vs. 5.7%, *P* = 0.001). For patients who survived to discharge, the three-year cumulative survival rates were 75.0% ± 8.2% and 81.2% ± 3.4% in the elderly and non-elderly groups, respectively (*P* = 0.484). In conclusion, elderly patients are at a higher risk of in-hospital mortality after surgery for LSNIE. However, once elderly patients are stabilized by surgical treatment and survive to discharge, the mid-term outcomes are promising.

## Introduction

Infective endocarditis (IE) is a serious infection associated with significant morbidity and mortality. In-hospital mortality rates have been reported as 15–28% in previous studies^1,2^. Although diagnostic tools, management algorithms, and antibiotic medications for IE are improving, IE treatment still remains a challenge for clinicians. For patients with uncontrolled infection or uncompensated heart failure, an early surgical approach is recommended to prevent progressive valvular structural damage and catastrophic systemic embolism, which are associated with poor prognosis^3–5^. The elderly population is increasing in developed countries worldwide^6^, and advanced age is an important risk factor for IE^7–9^. However, elderly patients tend to receive more conservative treatment strategies with a small proportion undergoing surgery, which was reported as only 38–47% in previous studies^10,11^. This retrospective study aimed to clarify the short- and mid-term outcomes among elderly patients who underwent cardiac valvular surgery for active left-sided native valve IE (LSNIE) based on an individual centre’s experience.

## Methods

### Patient enrolment and preoperative management

This study was approved by Chang-Gung medical foundation institutional ethics committee (No. 201801907B0). The need for informed consent was waived due to the retrospective nature of the study. Overall, 219 consecutive adult patients underwent cardiac surgery for left-sided IE at a single institution between July 2005 and September 2015.

Each patient fulfilled the modified Duke criteria for IE^12^, and surgical treatment was indicated based on published guidelines prior to 2015^13,14^. General cardiac function, valvular destruction severity, and vegetation size/location were assessed by an experienced cardiovascular physician using transthoracic or transoesophageal echocardiography. Active endocarditis was defined as definitive IE requiring intravenous antibiotic therapy until surgery^15^. After excluding 23 patients with sub-acute IE that resolved after a complete antibiotic treatment course and underwent delayed surgery due to residual valvular insufficiency found by follow-up echocardiography, and 17 with prosthetic valve IE, 179 patients were included. The patients were classified into two age groups: ≥65 years (elderly group, n = 38, 21.2%) and <65 years (non-elderly group, n = 141, 78.8%) according to previous definitions of elderly^10,16^. Preoperative septic emboli-related complications were diagnosed according to clinical symptoms and image studies, including computed tomography and magnetic resonance imaging, which were interpreted by experienced radiologists. If the vegetations were located in the aortic valve, preoperative coronary angiography was avoided to prevent new systemic embolic events. For patients with unstable haemodynamics or cardiopulmonary failure, depending on the inotropic agents and ventilator support, surgery was performed on an emergency basis.

### Surgical management

Intraoperative transoesophageal echocardiography was performed by specialized cardiovascular anaesthesiologists to confirm the preoperative diagnosis and extent of the valvular lesion. Using a standard sternotomy approach, cardiopulmonary bypass (CPB) was established via cannulation of the ascending aorta and right atrium or the venae cavae. Cardiac arrest was induced by a single dose of histidine–tryptophan–ketoglutarate solution or intermittent cold-blood cardioplegic solution through the coronary orifice. The diseased valves were carefully inspected, and the feasibility of valvular repair was considered after complete debridement of the infected tissues. The decision of valve repair or replacement was dependent on the surgeon’s discretion regarding the degree of valvular destruction and patient characteristics, including heart function, comorbidities, and preoperative haemodynamics. For infected mitral valves that were repairable, the Carpentier principle was applied^17^, and ring annuloplasty was used to ensure the long-term durability of repair. In Regards to infected aortic valves that were repairable, a pericardium patch was usually used for reconstructing the valvular defects following debridement. If valve replacement was necessary due to limited healthy tissue, the prosthetic choice was based on the patient’s individual preference after a detailed discussion before surgery. Before patients were weaned off CPB, haemostasis and good competency of the treated valve were confirmed, and a global assessment of cardiac function was performed.

### Postoperative care and interventions

After undergoing surgery for endocarditis, all patients were transferred to a specialized cardiovascular intensive care unit (ICU) for further treatment and observation. At 8 hours post-surgery, a ventilator-weaning protocol was initiated if there was no active bleeding, unstable haemodynamics, persistent arrhythmia, or signs of organ malperfusion. Renal replacement therapy was applied if acute renal failure developed after surgery, according to the Acute Kidney Injury Network criteria^18^. Further survey imaging, endovascular intervention, and surgical exploration for bleeding or malperfusion were performed, if indicated.

### Statistical analyses

Statistical analyses were performed using SPSS for Windows (version 22.0, IBM Corp., Armonk, NY). The Shapiro*–*Wilk test was used to test whether the continuous variables were normally distributed. Data were presented as means ± standard deviation for normally distributed variables, whereas medians and interquartile ranges were used to describe non-normally distributed data. The independent t test was performed for comparison of normally distributed data and the Mann–Whitney U-test was used for non-normally distributed data between the two study groups. Dichotomous variables are presented as numbers (n) and percentages (%). Univariate analyses of categorical variables were performed using the chi-square test, or Fisher’s exact test to determine inter-group differences, where appropriate. Preoperative and surgical variables found to be significant in the univariate analysis of in-hospital mortality were included in a multivariate logistic regression analysis to identify the independent predictors of in-hospital mortality. Numbers of events (in-hospital mortality) per covariate fell below four or five in the present study; therefore, we used penalization through data augmentation to perform multivariate logistic regression to avoid sparse data bias^19,20^. The Hosmer-Lemeshow test was used to evaluate the goodness-of-fit for the multivariate logistic regression model^21^. The Kaplan–Meier method was used to estimate 3-year cumulative survival, which was compared using the log-rank test. For all analyses, statistical significance was set at *P* < 0.05.

## Results

### Patient demographics

**Figure 1:**
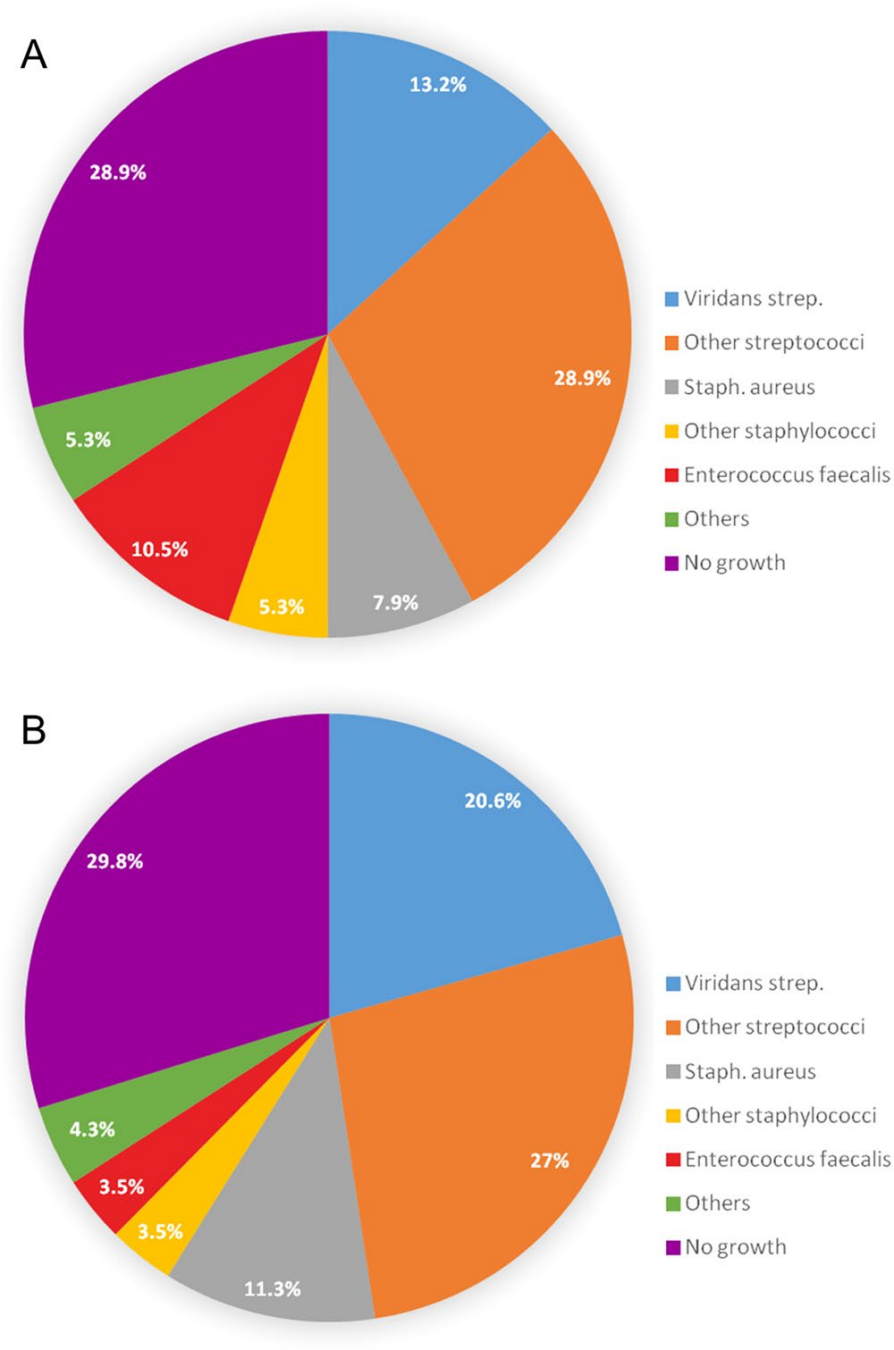
Distribution of microbial pathogens in the elderly group (**A**) and non-elderly group (**B**).

Table 1 shows the clinical demographics, comorbidities, preoperative conditions, and clinical presentation of the elderly and non-elderly groups. The average ages were 74.2 ± 6.4 and 45.2 ± 12.6 years in the elderly and non-elderly groups, respectively. Overall, 27.4% of patients were female, and diabetes mellitus was the most prevalent comorbidity, accounting for >20% of cases in both age groups, followed by hypertension and end-stage renal disease (ESRD). The average European System for Cardiac Operative Risk Evaluation (EuroSCORE) II estimated that mortality risk was higher in the elderly group (6.6% [4.1–11.7%] vs. 2.4% [1.8–4.1%]; *P* = 0.001) than in the non-elderly group. Bacteraemia was the most prevalent clinical finding, followed by fever and septic emboli-related complications in both the elderly and non-elderly groups. The elderly group had a lower incidence of septic emboli-related complications (23.7% vs. 42.6%; *P* = 0.034). There were no differences in the antibiotic courses and length of hospital stays before surgery between the elderly and non-elderly groups. The epidemiology of pathogens was generally similar, and streptococci accounted for >40% in both groups (Fig. 1).

**Table 1:** Preoperative characteristics according to the patient group.

Parameters	Overall	Elderly (≥65 years)	Non-elderly (<65 years)	*P-*value
	n = 179	n = 38	n = 141	
**Clinical demographics**				
Sex (female, n, %)	49, 27.4	14, 36.8	35, 24.8	0.140
Age (years)	51.3 ± 16.6	74.2 ± 6.4	45.2 ± 12.6	0.001
Body mass index (kg/m^2^)	21.5 (19.1–24.6)	22.7 (18.9–26.2)	21.5 (19.2–23.7)	0.174
Hypertension (n, %)	16, 8.9	5, 13.2	11, 7.8	0.304
Diabetes mellitus (n, %)	43, 24.0	11, 28.9	32, 22.7	0.423
Liver cirrhosis (n, %)	8, 4.5	0, 0	8, 5.7	0.133
Creatinine (mg/Dl)	1.1 (0.8–1.5)	1.3 (1.0–2.3)	1.0 (0.7–1.4)	0.001
eGFR (ml/min/1.73 m^2^)	77.3 (46.8–116.8)	49.2 (24.6–74.6)	86.6 (55.9–126.7)	0.001
ESRD (n, %)	16, 8.9	4, 10.5	12, 8.5	0.699
IV drug abuser (n, %)	7, 3.9	0, 0	7, 5.0	0.161
**Preoperative condition**				
Critical status (n, %)	31, 17.3	10, 26.3	21, 14.9	0.099
Ventilator support (n, %)	27, 15.1	9, 23.7	18, 12.8	0.095
Inotropic support (n, %)	12, 6.7	4, 10.5	8, 5.7	0.288
ICU hospitalization (n, %)	27, 15.1	8, 21.1	19, 13.5	0.247
LVEF (%)	65.4 ± 9.8	66.5 ± 10.6	65.1 ± 9.5	0.434
LVEF <50% (n, %)	12, 6.7	2, 5.3	10, 7.1	0.689
EuroSCORE II (%)	2.9 (1.8–6.1)	6.6 (4.1–11.7)	2.4 (1.8–4.1)	0.001
Antibiotics course before surgery (days)	15.0 (6.0–28.0)	14.5 (6.0–28.5)	15.0 (6.0–27.5)	0.712
Hospital stay before surgery (days)	20.0 (9.0–31.0)	19.0 (9.8–31.5)	20.0 (8.5–31.0)	0.609
**Clinical presentation**				
Fever (n, %)	117, 65.4	25, 65.8	92, 65.2	0.950
Malaise (n, %)	7, 3.9	1, 2.6	6, 4.3	0.647
Bacteraemia (n, %)	126, 70.4	27, 71.1	99, 70.2	0.920
Heart failure, NYHA-FC ≥ 3 (n, %)	28, 15.6	6, 15.8	22, 15.6	0.978
Septic emboli-related complications (n, %)	69, 38.5	9, 23.7	60, 42.6	0.034
Cerebral infarction (n, %)	41, 22.9	7, 18.4	33, 24.1	0.459
ICH (n, %)	12, 6.7	0, 0	12, 8.5	0.063
Cerebral mycotic aneurysm (n, %)	8, 4.5	0, 0	8, 5.7	0.133
Others^a^ (n, %)	20, 11.2	2, 5.3	18, 12.8	0.193
Recurrent thromboembolism (n, %)	9, 5.0	1, 2.6	8, 5.7	0.446

^a^Splenic infarction in 12; renal infarction in 4; spinal abscess in 2; liver abscess in 1; and lower limbs ischemia in 1.

ESRD, end-stage renal disease; EuroSCORE, European System for Cardiac Operative Risk Evaluation; ICH, intracranial haemorrhage; ICU, intensive care unit; IV, intravenous; LVEF, left ventricular ejection fraction; NYHA-FC, New York heart association*-*functional classification.

### Preoperative echocardiographic assessment and surgical information

Table 2 provides detailed information regarding preoperative echocardiographic assessment and surgical variables. In general, there were no differences in the location of the diseased valves, size/number of vegetations, and occurrence of periannular abscess between the groups. Overall, 23.5% of the patients underwent emergency surgery. Regarding the valvular procedures, the rates of repair and replacement were similar in the elderly and non-elderly groups. However, more patients in the non-elderly group underwent mechanical valve replacement for both the aortic and mitral valve. The time span of CPB, aortic cross-clamp, and rate of histidine-tryptophan-ketoglutarate cardioplegic solution usage did not differ between the two groups. In total, 11.7% of patients underwent extracorporeal membrane oxygenation installation in the operating room due to intraoperative myocardial failure.

**Table 2:** Echocardiographic assessment and surgical information according to the patient group.

Parameters	Overall	Elderly (≥65 years)	Non-elderly (<65 years)	*P-*value
	n = 179	n = 38	n = 141	
**Echocardiographic assessment**				
Maximal vegetation diameter (mm)	13.0 (8.0–18.0)	11.0 (8.5–16.5)	13.8 (8.0–18.0)	0.249
Multiple vegetation (n, %)	48, 26.8	7, 18.4	41, 29.1	0.188
Severe aortic regurgitation (n, %)	66, 36.9	11, 28.9	55, 39.0	0.254
Severe mitral regurgitation (n, %)	73, 40.8	19, 50.0	54, 38.3	0.193
Severe aortic + mitral regurgitation (n, %)	22, 12.3	5, 13.2	17, 12.1	0.854
Periannular abscess (n, %)	12, 6.7	1, 2.6	11, 7.8	0.258
**Surgical information**				
Emergency surgery (n, %)	42, 23.5	9, 23.7	33, 23.4	0.971
Aortic valve repair (n, %)	5, 2.8	1, 2.6	4, 2.8	0.946
Aortic valve replacement (n, %)	84, 46.9	19, 50.0	65, 46.1	0.699
Tissue valve (n, %)	65, 36.3	19, 50.0	46, 32.6	0.048
Mechanical valve (n, %)	19, 10.6	0, 0	19, 13.5	0.017
Mitral valve repair (n, %)	75, 41.9	15, 39.5	60, 42.6	0.733
Mitral valve replacement (n, %)	63, 35.2	14, 36.8	49, 34.8	0.811
Tissue valve (n, %)	49, 27.4	14, 36.8	35, 24.8	0.140
Mechanical valve (n, %)	14, 7.8	0, 0	14, 9.9	0.043
Tricuspid annuloplasty (n, %)	19, 10.6	4, 10.5	15, 10.6	0.984
Tricuspid valve replacement (n, %)	1, 0.6	1, 2.6	1, 0	0.053
CABG (n, %)	4, 2.2	3, 7.9	1, 0.7	0.008
Maze procedure (n, %)	4, 2.2	3, 7.9	1, 0.7	0.008
Cardiopulmonary bypass time (min)	147.0 (118.0–182.0)	156.5 (120.3–203.5)	146.0 (115.5–176.5)	0.335
Aortic clamping time (min)	107.0 (81.0–132.0)	105.5 (75.5–125.0)	107.0 (81.5–132.0)	0.489
HTK cardioplegic solution (n, %)	38, 21.2	8, 21.1	30, 21.3	0.976
ECMO support (n, %)	21, 11.7	5, 13.2	16, 11.3	0.758
IABP support (n, %)	9, 5.0	3, 7.9	6, 4.3	0.362

CABG, coronary artery bypass graft; ECMO, extracorporeal membrane oxygenation; HTK, histidine-tryptophan-ketoglutarate; IABP, intra-aortic balloon pump.

### Postoperative complications

As shown in Table 3, a significantly higher in-hospital mortality rate was observed in the elderly group compared to the non-elderly group (26.3% vs. 5.7%; *P* = 0.001). Moreover, the elderly group showed a higher incidence of complications, including new cerebral infarction, prolonged ventilator dependence, and prolonged ICU course. The median hospital stays were 37.5 (16.8–53.0) and 37.0 (28.0–47.5) days in the elderly and non-elderly groups, respectively.

**Table 3:** Postoperative mortality and morbidity according to the patient group.

Parameters	Overall	Elderly (≥65 years)	Non-elderly (<65 years)	*P-*value
	n = 179	n = 38	n = 141	
Hospital mortality^a^ (n, %)	18, 10.1	10, 26.3	8, 5.7	0.001
Myocardial failure^b^ (n, %)	9, 5.0	7, 18.4	2, 1.4	
Brain stem failure^c^ (n, %)	4, 2.2	1, 2.6	3, 2.1	
Uncontrolled sepsis^d^ (n, %)	5, 2.8	2, 5.3	3, 2.1	
Acute renal failure^e^ (n, %)	8, 4.5	2, 5.3	6, 4.3	0.790
Atrial fibrillation (n, %)	22, 12.3	5, 13.2	17, 12.1	0.854
GI bleeding (n, %)	5, 2.8	2, 5.3	3, 2.1	0.298
Deep sternal wound infection (n, %)	6, 3.4	2, 5.3	4, 2.8	0.461
Pneumonia (n, %)	17, 9.5	4, 10.5	13, 9.2	0.807
New brain stroke (n, %)	11, 6.1	5, 13.2	6, 4.3	0.043
Infarction (n, %)	5, 2.8	3, 7.9	2, 1.4	0.032
Haemorrhage (n, %)	7, 3.9	2, 5.3	5, 3.5	0.628
Re-operation for bleeding (n, %)	10, 5.6	4, 10.5	6, 4.3	0.135
Extubation time (h)	20.0 (8.0–48.0)	41.0 (17.8–70.5)	19.0 (6.5–44.0)	0.001
Ventilator support >48 h (n, %)	45, 25.1	17, 44.7	28, 19.9	0.002
Tracheostomy (n, %)	4, 2.2	2, 5.3	2, 1.4	0.155
ICU stay (days)	3.0 (2.0–5.0)	4.5 (2.8–9.0)	3.0 (2.0–4.0)	0.001
ICU stay >7 days (n, %)	29, 16.2	13, 34.2	16, 11.3	0.001
ICU readmission (n, %)	12, 6.7	4, 10.5	8, 5.7	0.288
Hospital stay (days)	37.0 (27.0–49.0)	37.5 (16.8–53.0)	37.0 (28.0–47.5)	0.405

^a^Death occurring during hospitalization.

^b^Persistent myocardial dysfunction with shock, refractory to inotropic medication and/or mechanical support.

^c^Cerebral infarction/haemorrhage with irreversible deep coma, apnoea, and absence of brainstem reflexes.

^d^Persistent bacteraemia, refractory to antibiotic and surgical treatments.

^e^Stage 3 acute kidney dysfunction according to the Acute Kidney Injury Network classification.

GI, gastrointestinal; ICU, intensive care unit.

### Regression analysis of in-hospital mortality

Table 4 shows the regression analysis results among patients in the elderly group, including female, ESRD, emergency surgery, preoperative inotropic support, preoperative ICU hospitalization, and left ventricular ejection fraction. Four significant prognostic factors for in-hospital mortality were identified: female sex (adjusted odds ratio (aOR) 4.45; 95% confidence interval (CI) 1.46–13.72; *P* = 0.009), ESRD (aOR 7.02; 95% CI 2.07–23.85; *P* = 0.002), emergency surgery (aOR 3.73; 95% CI 1.16–11.93; *P* = 0.027), and preoperative ICU hospitalization (aOR 4.51; 95% CI 1.38–14.74; *P* = 0.013). The Hosmer-Lemeshow test showed an acceptable goodness-of-fit for the multivariate logistic regression model (*P* = 0.844).

**Table 4:** Logistic regression analyses for hospital mortality of elderly group.

Parameters	β-coefficient	Standard error	Odds ratio, 95% CI	*P-*value	Adjusted for sparse data bias
**Univariate logistic regression**					
Female	1.946	0.816	7.00 (1.41–34.68)	0.017	
ESRD	2.449	1.230	11.57 (1.04–128.97)	0.047	
Emergency surgery	1.792	0.832	6.00 (1.18–30.62)	0.031	
Preoperative inotropic support	2.449	1.230	11.57 (1.04–128.97)	0.047	
Preoperative ICU hospitalization	2.120	0.879	8.33 (1.49–46.71)	0.016	
LVEF	0.092	0.045	1.10 (1.00–1.20)	0.042	
**Multivariate logistic regression**^a^					
Female	2.205	1.357	9.07 (0.64–129.55)	0.104	Before
	1.493	0.570	4.45 (1.46–13.72)	0.009	After
ESRD	4.620	1.813	104.49 (2.91–3544.27)	0.011	Before
	1.949	0.622	7.02 (2.07–23.85)	0.002	After
Emergency surgery	1.640	1.662	5.15 (0.20–133.81)	0.774	Before
	1.316	0.594	3.73 (1.16–11.93)	0.027	After
Preoperative inotropic support	–0.024	2.345	0.98 (0.01–96.69)	0.992	Before
	0.060	0.642	1.06 (0.30–3.76)	0.926	After
Preoperative ICU hospitalization	2.625	1.778	13.80 (0.42–450.19)	0.140	Before
	1.506	0.604	4.51 (1.38–14.74)	0.013	After
LVEF	0.019	0.066	1.02 (0.90–1.16)	0.774	Before
	0.026	0.047	1.03 (0.94–1.14)	0.575	After

^a^Hosmer-Lemeshow test, *P* = 0.844.

ESRD, end-stage renal disease; ICU, intensive care unit; LVEF, left ventricular ejection fraction.

### Cumulative survival and left ventricular function at 3 years

**Figure 2:**
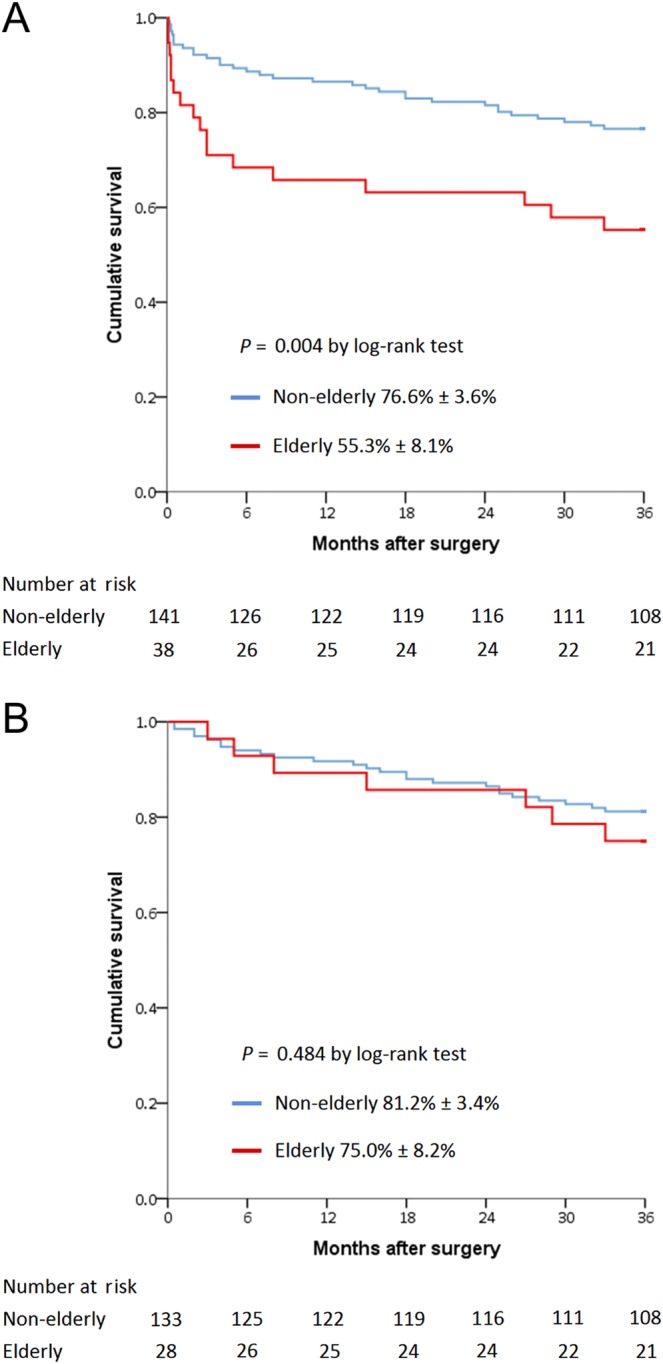
Three-year cumulative survival rates for 179 patients including in-hospital mortality (**A**) and for 161 patients excluding in-hospital mortality (**B**) stratified by age.

For the overall patient cohort, the elderly group had an inferior 3-year cumulative survival rate (Fig. 2A). However, for patients who survived to discharge (Fig. 2B), the 3-year cumulative survival curves were not significantly different between the elderly and non-elderly groups (75.0 ± 8.2% vs. 81.2 ± 3.4%; *P* = 0.484).

## Discussion

An increasing number of elderly patients suffer from cardiovascular diseases, including IE, which can be life-threatening^7–9^. However, elderly patients tend to receive a more conservative treatment course due to concerns regarding surgical complications^10,11^. In this single-centre study, a comparative cohort of patients who underwent surgical treatment for active LSNIE is presented, which includes 38 elderly patients aged >65 years. In-hospital mortality rates and postoperative complication rates were higher in this population than in non-elderly patients. However, the mid-term outcomes were still satisfactory for elderly patients who survived to hospital discharge.

Due to the increase in average life expectancy and the higher incidence of cardiovascular disease with advancing age, more elderly patients nowadays present for cardiac surgery^22^. However, advanced age is considered a predictor of adverse outcomes following cardiac surgery^23,24^. An increased but acceptable surgical mortality rate has been previously observed among elderly patients with IE^11,25^, which was also observed in our study. Therefore, surgery may be justified as a treatment option for elderly patients with LSNIE and should be considered in selected patients. Thorough consultation between the medical and surgical teams is essential to make this a reliable strategy.

Traditional surgery with CPB remains the standard treatment option for IE. However, the aging process is associated with structural and functional changes in various organ systems, possibly reducing the tolerance for haemodynamic fluctuation, and increasing coagulopathy and the systemic inflammatory response induced by CPB. Therefore, higher in-hospital mortality and complication rates are expected in this population. In the present study, the elderly group demonstrated a higher EuroSCORE II-estimated risk, which also addresses the inferior short-term outcome. The aging process is associated with numerous ionic, molecular and biochemical changes in the heart^22,26^. These age-related changes can affect cardiac morphology and reduce the physiologic function. Therefore, cardiac aging results in decreased mechanical and contractile efficiency, and may increase the rate of cardiomyocyte apoptosis after cardiac surgery. In our study, approximately 20% of elderly patients required mechanical support following surgery, and >60% of the mortality in the elderly group was associated with postoperative myocardial failure. Although the rates of postoperative mechanical support were similar in the two groups, the aging heart may exhibit inferior recovery compared with younger hearts.

Septic emboli-related complications occur in 20–50% of patients with IE, especially when the diseased valve is on the left side^27,28^. The central nervous system is the most common destination of embolism, followed by the spleen, kidneys, lungs, and liver. In our study, the elderly group had a relatively lower incidence of preoperative septic emboli-related complications than the non-elderly group. We suggest several reasons for this finding. First, contrast-enhanced imaging may have been arranged more conservatively for patients in the elderly group due to the lower reserved renal function. Second, the symptoms and presentations of emboli-related complications in the elderly may not be apparent and, therefore, could have been overlooked. When the emboli-related complications have progressed to a serious stage, the timing of surgical treatment may be delayed, and the outcome is compromised. Finally, no patients in the elderly group experienced preoperative intracranial haemorrhage or cerebral mycotic aneurysm. These two cerebral complications could increase the surgical risk and induce permanent neurologic deficits. According to previous studies^15,29^, 13% of hospital mortality and 24% of postoperative cardiac and cerebrovascular complications were reported in this high-risk subgroup. As these complications are extremely serious and life-threatening, elderly patients with these conditions may be reluctant to undergo surgery.

The elderly population is associated with a high incidence of comorbidities, including chronic kidney disease (CKD), and relative risk factors, including hypertension and diabetes, which predispose them to CKD^30^. According to a previous multicentre study^31^, patients with CKD had an increased perioperative mortality rate and reduced long-term survival after cardiac surgery. In our study, the elderly group showed worse renal function compared to the non-elderly group before surgery (49.2 (24.6–74.6) vs. 86.6 (55.9–126.7) ml/min/1.73 m^2^; *P* = 0.001). Furthermore, ESRD was one of the independent predictors of in-hospital mortality for the elderly group. The mechanism underlying the negative impact of CKD on cardiac surgery may be multifactorial, including perioperative electrolyte/fluid imbalance, compromised immunity, prevalent systemic vascular disease, and poor tolerance to acute kidney injury induced by severe infection or surgical procedures. For patients with ESRD, these influences can be magnified. Therefore, we suggest that elderly patients with CKD who present with clinical signs of IE should undergo early assessments and receive careful surgical management to optimize the outcomes.

Elderly patients who underwent surgery for IE were associated with higher in-hospital mortality and morbidity rates owing to their comorbid conditions and delayed timing of surgery. However, the late surgical outcomes are rarely reported. Similar short-term results were presented in our study. Furthermore, elderly patients who survived to discharge could have a comparable 3-year survival to that of the non-elderly group. Therefore, we suggest that a guideline-directed surgical strategy according to the presence of complications, which include embolism events, large vegetation, heart failure, or uncontrolled infection, would be beneficial to improve the mid-term outcomes of elderly patients with LSNIE.

### Limitations of this study

Our study had several important limitations. First, because the study used a retrospective and nonrandomized control design with a small sample size, bias might exist that influenced the homogeneity of the study groups and the stability of the multivariate logistic regression model. Second, as this cohort spanned a period of >10 years, the technology of CPB and myocardial protection, as well as strategies for treating IE and ICU care protocols may have changed over this time period. Third, as this was a retrospective study, some haemodynamic data, laboratory profiles, and inotropic medication dosage information were not completely analysed due to incomplete records. This hindered more detailed analyses of physiological fluctuations during the perioperative course. Finally, despite the satisfactory mid-term results, an extended follow-up study should be conducted in the future to evaluate the long-term outcomes in the elderly population.

## Conclusions

Even with similar demographics and surgical procedures, elderly patients are at higher risk of in-hospital mortality and complications after surgery for active LSNIE than non-elderly patients. However, if such patients are stabilized by surgical treatment and able to survive to discharge, the mid-term outcomes can still be comparable to that of the younger population. Accurate surgical management planning and careful assessment before disease progression are mandatory to improve outcomes.

## Data Availability

All data generated or analysed in this study are available from the corresponding author on reasonable request.

## References

[1] Leone S (2012). Epidemiology, characteristics, and outcome of infective endocarditis in Italy: the Italian study on endocarditis. Infection..

[2] Olmos C (2013). Contemporary epidemiology and prognosis of septic shock in infective endocarditis. Eur. Heart J..

[3] Habib G (2015). 2015 ESC Guidelines for the management of infective endocarditis: the Task Force for the Management of Infective Endocarditis of the European Society of Cardiology (ESC). Endorsed by: European Association for Cardio-Thoracic Surgery (EACTS), the European Association of Nuclear Medicine (EANM). Eur. Heart J..

[4] Kang DH (2012). Early surgery versus conventional treatment for infective endocarditis. N. Engl. J. Med..

[5] Sorabella RA (2015). Early operation for endocarditis complicated by preoperative cerebral emboli is not associated with worsened outcomes. Ann. Thorac. Surg..

[6] Christensen K, Doblhammer G, Rau R, Vaupel JW (2009). Ageing populations: the challenges ahead. Lancet..

[7] Steckelberg JM (1990). Influence of referral bias on the apparent clinical spectrum of infective endocarditis. Am. J. Med..

[8] Fefer P, Raveh D, Rudensky B, Schlesinger Y, Yinnon AM (2002). Changing epidemiology of infective endocarditis: a retrospective survey of 108 cases, 1990-1999. Eur. J. Clin. Microbiol. Infect. Dis..

[9] Von Reyn CF, Levy BS, Arbeit RD, Friedland G, Crumpacker CS (1981). Infective endocarditis: an analysis based on strict case definitions. Ann Intern. Med..

[10] Durante-Mangoni E (2008). Current features of infective endocarditis in elderly patients: results of the International Collaboration on Endocarditis Prospective Cohort Study. Arch. Intern. Med..

[11] López J (2010). Age-dependent profile of left-sided infective endocarditis: a 3-center experience. Circulation..

[12] Li JS (2000). Proposed modifications to the Duke criteria for the diagnosis of infective endocarditis. Clin. Infect. Dis..

[13] Baddour LM (2005). Infective endocarditis: diagnosis, antimicrobial therapy, and management of complications: a statement for healthcare professionals from the committee on rheumatic fever, endocarditis, and Kawasaki disease, council on cardiovascular disease in the young, and the councils on clinical cardiology, stroke, and cardiovascular surgery and anesthesia, American Heart Association: endorsed by the infectious diseases society of America. Circulation..

[14] Nishimura RA (2014). 2014 AHA/ACC guideline for the management of patients with valvular heart disease: executive summary: a report of the American College of Cardiology/American Heart Association task force on practice guidelines. Circulation..

[15] Yoshioka D (2014). Valve surgery in active endocarditis patients complicated by intracranial haemorrhage: the influence of the timing of surgery on neurological outcomes. Eur. J. Cardiothorac Surg..

[16] Gagliardi JP (1998). Native valve infective endocarditis in elderly and younger patients: comparison of clinical features and outcomes with use of Duke criteria and the Duke endocarditis database. Clin. Infect. Dis..

[17] Carpentier, A., Adams, D. H. & Filsoufi, F. Carpentier’s Reonstructive Valve Surgery. Philadelphia, PA: Saunders *Elsevier* 27–171 (2010).

[18] Mehta RL (2007). Acute kidney injury network: report of an initiative to improve outcomes in acute kidney injury. Crit. Care..

[19] Peduzzi P, Concato J, Kemper E, Holford TR, Feinstein AR (1996). A simulation study of the number of events per variable in logistic regression analysis. J Clin Epidemiol..

[20] Greenland S, Mansournia MA, Altman DG (2016). Sparse data bias: A problem hiding in plain sight. BMJ..

[21] Hosmer DW, Lemeshow S (1980). A goodness-of-fit test for the multiple logistic regression model. Commun Stat..

[22] Nicolini F (2014). The evolution of cardiovascular surgery in elderly patient: a review of current options and outcomes. BioMed. Res. Int..

[23] Naughton C, Feneck RO, Roxburgh J (2009). Early and late predictors of mortality following on-pump coronary artery bypass graft surgery in the elderly as compared to a younger population. Eur. J. Cardiothorac. Surg..

[24] Edwards MB, Taylor KM (2003). Outcomes in nonagenarians after heart valve replacement operation. Ann. Thorac. Surg..

[25] Remadi JP (2009). Infective endocarditis in elderly patients: clinical characteristics and outcome. Eur. J. Cardiothorac. Surg..

[26] Folkow B, Svanborg A (1993). Physiology of cardiovascular aging. Physiol. Rev..

[27] Mouly S (2002). The changing clinical aspects of infective endocarditis: descriptive review of 90 episodes in a French teaching hospital and risk factors for death. J. Infect..

[28] Thuny F (2005). Risk of embolism and death in infective endocarditis: prognostic value of echocardiography, a prospective multicenter study. Circulation..

[29] Okita Y (2016). Optimal timing of surgery for active infective endocarditis with cerebral complications: a Japanese multicentre study. Eur. J. Cardiothorac. Surg..

[30] Mallappallil M, Friedman EA, Delano BG, McFarlane SI, Salifu MO (2014). Chronic kidney disease in the elderly: evaluation and management. Clin. Pract. (Lond)..

[31] Fernando M (2014). Outcomes of cardiac surgery in chronic kidney disease. J. Thorac. Cardiovasc. Surg..

